# Incidence of Pleural Recurrence after Computed Tomography-Guided Needle Biopsy in Stage I Lung Cancer

**DOI:** 10.1371/journal.pone.0042043

**Published:** 2012-08-02

**Authors:** Keisuke Asakura, Yotaro Izumi, Yoshikane Yamauchi, Seishi Nakatsuka, Masanori Inoue, Hideki Yashiro, Takayuki Abe, Yuji Sato, Hiroaki Nomori

**Affiliations:** 1 Division of General Thoracic Surgery, Department of Surgery, School of Medicine, Keio University, Tokyo, Japan; 2 Department of Diagnostic Radiology, School of Medicine, Keio University, Tokyo, Japan; 3 Centre for Clinical Research, School of Medicine, Keio University, Tokyo, Japan; National Taiwan University Hospital, Taiwan

## Abstract

**Objective:**

A risk of tumor seeding after percutaneous needle biopsy has been reported in various organs, including the lung. This study retrospectively evaluated the proportion of ipsilateral pleural recurrence after computed tomography-guided needle biopsy (CTNB) in p-stage I lung cancer patients.

**Methods:**

Of the 321 patients diagnosed with p-stage I lung cancer, 124 underwent CTNB before surgery, while 197 underwent non-CTNB procedures, including bronchoscopic biopsy in 188 patients and thoracoscopic wedge resection in 9. These patients were retrospectively analyzed.

**Results:**

While the tumor size was significantly larger in the non-CTNB group (25±9 mm) in comparison to the CTNB group (19±9 mm) (p<0.001), percentage of pleural, vascular, or lymphatic invasions were comparable between the two groups. Eight patients developed ipsilateral pleural recurrences, one (1%) in the CTNB group, and 7 (4%) in the non-CTNB group. Of these, 3 patients developed pleural recurrence only at first, 1 (1%) in the CTNB group, and 2 (1%) in the non-CTNB group. The differences in the proportions of these pleural recurrences between the 2 groups were not significant. Subgroup analyses by baseline characteristics such as tumor size, pT stage, or microscopic pleural invasion, showed that proportions of pleural recurrences in CTNB group were not high compared with non-CTNB group in each subgroup. Analysis of progression-free survival showed that recurrences in CTNB were not high compared with non-CTNB.

**Conclusions:**

The pleural recurrence was not significantly increased after CTNB in p-stage I lung cancer patients in this particular study.

## Introduction

Recent advances in diagnostic imaging using high-resolution CT have enabled the visualization of small lung nodules. Although bronchoscopic biopsy is one of the commonly used methods for obtaining a confirmed diagnosis, it is not always suitable for diagnosing small lung lesions because of the difficulty of detecting and hitting the nodules under plain chest fluoroscopy [1]. Recently, percutaneous core-needle lung biopsy under multi-CT fluoroscopic guidance (CTNB) has been reported to be an effective procedure with increased diagnostic accuracy compared with other conventional methods for the diagnosis of small lung nodules [2]–[4]. A large-scale study extracted from the Surveillance, Epidemiology, and End Results Registry in 2006 showed no overall increases in cancer-related deaths in lung cancer patients who had undergone transthoracic needle biopsies [5], but the actual patterns of recurrences are not reported in the study. Tumor implantations have been reported after CTNB [6]–[9], and some reports have suggested an increased incidence of pleural recurrence even in early-stage lung cancer patients after CTNB [10], [11]. Therefore, in the present study, we retrospectively investigated the incidence of postoperative pleural recurrence and progression-free survival (PFS) in resected p-stage I lung cancer patients who underwent CTNB for diagnosis at our institution. The results were also compared with resected p-stage I lung cancer patients who did not undergo CTNB for diagnosis (non-CTNB).

**Table 1:** Patient demographics.

	Groups		
	CTNB	non-CTNB	p Value
Total	124	197	
Sex			
Male	69 (56)	129 (66)	0.08
Female	55 (44)	68 (35)	
Age (years)	65±10	66±11	0.3
Histology			
Adenocarcinoma	112 (90)	161 (82)	0.04
Squamous cell carcinoma	9 (7)	28 (14)	0.06
Others	3 (2)	8 (4)	0.4
Tumor size (mm)	19±9	25±9	<0.001
T factor			
pT1a	79 (64)	78 (40)	<0.001
pT1b	25 (20)	51 (26)	0.24
pT2a	20 (16)	68 (35)	<0.001
Surgical procedure			
Lobectomy or more	111 (90)	186 (94)	0.1
Sublobar resection	13 (10)	11 (6)	
Microscopic pleural invasion			
yes	12 (10)	23 (12)	0.6
no	112 (90)	174 (88)	
Microscopic vascular invasion			
yes	7 (6)	19 (10)	0.3
no	117 (94)	178 (90)	
Microscopic lymphatic invasion			
Yes	12 (10)	24 (12)	0.5
no	112 (90)	173 (88)	

The numbers in parentheses indicate percentages.

## Methods

### Patients

Between October 2002 and February 2009, 582 patients with lung cancer underwent surgical resection at our institution. Of these, 321 patients were diagnosed with pathologic stage I disease, and they were reviewed retrospectively. The institutional review board, Keio University School of Medicine an Ethical Committee, approved the study and waived the need for individual patient consent because this is a retrospective study and consent cannot be obtained from all patients, and also because individual patients are not identified in the study. This is in accordance with the Ethical Guidelines for Clinical Studies published by the Japanese Ministry of Health, Labour and Welfare.

**Table 2:** Type of recurrences.

	Groups		
	CTNB	non-CTNB	p Value
Total	124	197	
Recurrences	11 (9)	35 (18)	0.03
Site of the recurrences			
Distant	8 (7)	28 (14)	0.03
Local	4 (3)	18 (9)	0.04
Both	1 (1)	11 (6)	0.03
Pleural recurrences	1 (1)	7 (4)	0.16
Pleural recurrences alone	1 (1)	2 (1)	1.00

The numbers in parentheses indicate percentages.

### Diagnostic Procedures

We attempted to obtain pretreatment diagnoses for all nodules, because we considered that non-surgical treatment options such as stereotactic radiation should be offered to these patients. This is particularly so in patients who are considered to be stage I. Diagnoses of patients with pulmonary lesions were carried out as follows. First, histological or cytological diagnoses using fiberoptic bronchoscopy were planned. If this was considered to be difficult or was performed but failed, the patients were then scheduled for CTNB. CTNB was performed using a multidetector CT (Aquilion 64; Toshiba Med. Co. Ltd., Tokyo, Japan). This device enables the use of three-slice simultaneous CT fluoroscopy. In all patients, the biopsy procedure was carried out in various postures, depending on the location of the lesion. The insertion route was intentionally chosen to include the lung parenchyma en route to the lesion, because it was considered that this may decrease the risk of tumor implantation at the pleura. All biopsy specimens were obtained using an 18-gauge core biopsy needle (Super-Core II TM, MD Tech, Fl, USA). The end-point of the procedure was confirmation by CT fluoroscopy that the needle hit the lesion on the 2 adjacent images above and below as well as on the central image, as well as the actual acquisition of macroscopic tumor tissue. If CTNB was considered to be difficult or the diagnosis was still undetermined after CTNB, a diagnostic wedge resection under thoracoscopy was conducted. Although diagnosis was not made by CTNB in these patients, these patients were included in the CTNB group because they had undergone CTNB.

**Figure 1:**
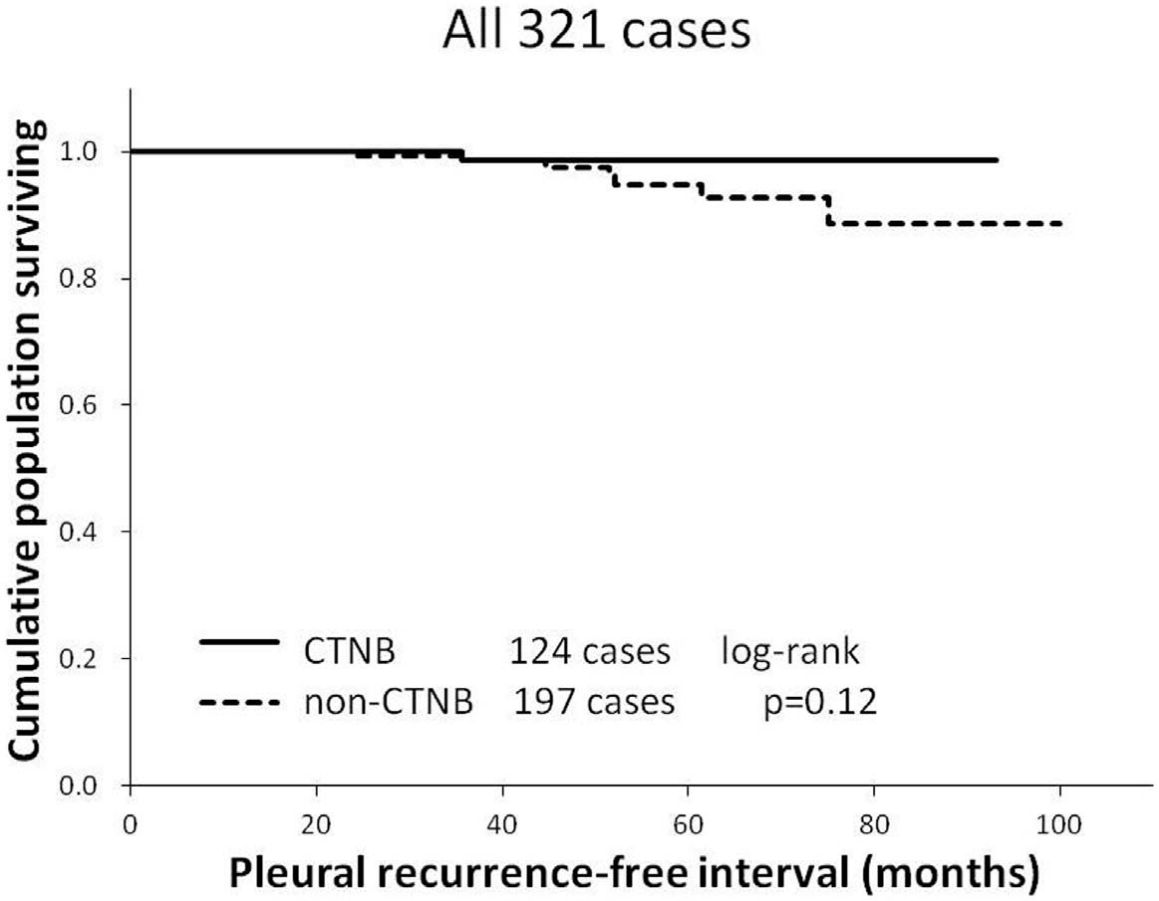
The pleural recurrence free interval in stage I lung cancer patients who underwent computed tomography-guided needle biopsy (CTNB group) versus patients who did not (non-CTNB group) is shown as a Kaplan-Meier estimate. There was no significant difference between the two groups (p = 0.12, log-rank test).

### Postoperative Follow-up

Pathological staging was classified according to the Staging Manual in Thoracic Oncology by the International Association for the Study of Lung Cancer, 2009 [12]. The guideline for postoperative follow-up is yet to be established in Japan, and currently at our institution, contrast-enhanced chest-abdominal-pelvic CT and contrast-enhanced brain MRI were routinely performed every 6 months to identify any recurrences for at least 5 years after surgery. Positron emission tomography/CT was used in some recent cases to support the diagnosis. Local recurrences were defined as any recurrences within the ipsilateral thorax. Pleural recurrence was defined as progressive growth and/or increase of pleural nodules or malignant pleural effusion proven by cytology.

**Table 3:** Proportions of pleural recurrence by each subgroup (p-T factor, tumor size, pleural invasion and histology).

	Groups		
	CTNB	non-CTNB	p Values
pT1a	79	78	
recurrence	5 (6)	7 (9)	0.56
pleural recurrence	0 (0)	1 (1)	0.50
pleural recurrence alone	0 (0)	0 (0)	1.00
pT1b	25	51	
recurrence	2 (8)	8 (16)	0.48
pleural recurrence	0 (0)	2 (4)	1.00
pleural recurrence alone	0 (0)	1 (2)	1.00
pT2a	20	68	
recurrence	4 (20)	20 (29)	0.57
pleural recurrence	1 (5)	4 (6)	1.00
pleural recurrence alone	1 (5)	1 (2)	0.40
Size = 0–20 mm	86	85	
recurrence	7 (8)	9 (11)	0.61
pleural recurrence	1 (1)	3 (4)	0.37
pleural recurrence alone	1 (1)	1 (1)	1.00
Size = 21–30 mm	26	61	
recurrence	2 (8)	10 (16)	0.50
pleural recurrence	0 (0)	2 (3)	1.00
pleural recurrence alone	0 (0)	1 (2)	1.00
Size = 31–50 mm	12	51	
recurrence	2 (17)	16 (29)	0.48
pleural recurrence	0 (0)	2 (4)	1.00
pleural recurrence alone	0 (0)	0 (0)	1.00
Microscopic pleural invasion (+)	12	23	
recurrence	2 (17)	5 (22)	1.00
pleural recurrence	1 (8)	3 (13)	1.00
pleural recurrence alone	1 (8)	1 (4)	1.00
Microscopic pleural invasion (-)	112	174	
recurrence	9 (8)	30 (17)	0.03
pleural recurrence	0 (0)	4 (2)	0.16
pleural recurrence alone	0 (0)	1 (1)	1.00
Adenocarcinoma	112	161	
recurrence	9 (8)	28 (17)	0.07
pleural recurrence	1 (1)	6 (4)	0.25
pleural recurrence alone	1 (1)	2 (1)	1.00
Other histological types	12	36	
recurrence	2 (17)	7 (19)	1.00
pleural recurrence	0 (0)	1 (3)	1.00
pleural recurrence alone	0 (0)	0 (0)	1.00

The numbers in parentheses indicate percentages.

### Statistical Analyses

For the analyses, patients who received CTNB at any point before surgery were included in the CTNB group. All other patients were included in the non-CTNB group. Baseline characteristics were summarized by each group. Homogeneity of those factors between the two groups was tested with chi-square test, Fisher's exact test or the Mann-Whitney U test. The proportion of recurrence was compared between the two groups with Fisher’s exact test. Progression-free survival (PFS) curves were estimated with the Kaplan-Meier method and were compared with the log-rank test. Significance level for all tests was two-sided, at 5%. All data were analyzed using IBM SPSS Statistics 19 software (IBM Corporation, USA).

**Table 4:** Clinicopathologic characteristics of 8 patients with pleural recurrences.

PatientNo.	Age/Sex	DiagnosticMethods	OperativeProcedure	Pathologic findings			ConcomitantRecurrences	Time toRecurrence (mo)	Outcome
				Histology	Size(mm)	pl/v/ly			
1	63/M	CTNB	Lobe	Ad	13	+/−/−	-	24	DOD
2	75/M	BFS	Lobe	Ad	20	+/+/+	-	10	DOD
3	83/F	BFS	Lobe	Ad	22	−/−/−	-	50	DOD
4	64/M	BFS	Lobe	Ad	12	+/+/−	LN, PUL	24	DOD
5	72/M	BFS	Lobe	Ad	15	−/−/−	PUL	36	DOD
6	75/M	BFS	Lobe	Sq	25	−/−/+	LN, PUL, HEP	50	DOD
7	53/M	BFS	Lobe	Ad	32	−/−/−	LN, PUL, BRA	36	DOD
8	69/M	BFS	Lobe	Ad	38	+/−/−	PUL	24	AWD

M = male, F = female, CTNB = computer tomography guided needle biopsy, BFS = bronchoscopy, Lobe = lobectomy, Ad = adenocarcinoma, Sq = squamous cell carcinoma, pl = microscopic pleural invasion, v =  microscopic vascular invasion, ly =  microscopic lymphatic invasion.

LN = lympho node, PUL = pulmonary metastasis, BRA = brain metastasis, HEP =  Liver metastasis, AWD = alive with disease, DOD = dead of disease.

## Results

### Patients

Patient demographics in the CTNB and non-CTNB groups are summarized in Table 1. There were 124 patients in the CTNB group and 197 patients in the non-CTNB group. The non-CTNB group included 188 patients who underwent bronchoscopic biopsy and 9 who underwent thoracoscopic wedge resections. In the CTNB group, the biopsy specimen was non-diagnostic in 6 patients (5%). These patients were diagnosed intraoperatively by wedge resection, and subsequently underwent lobectomy.

**Figure 2:**
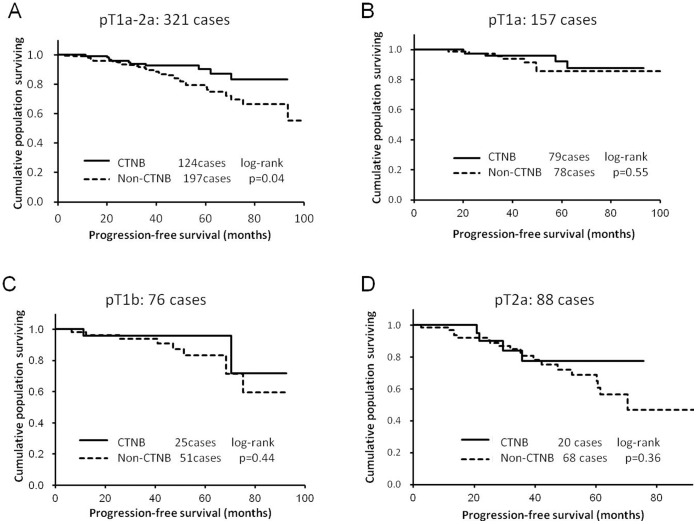
Progression-free survival (PFS) rates in stage I lung cancer patients who underwent computed tomography-guided needle biopsy (CTNB group) versus patients who did not (non-CTNB group). (A) PFS was significantly reduced in the non-CTNB group in comparison to the CTNB group overall (CTNB group, n = 124; non-CTNB group, n = 197; p = 0.04, log-rank test). (B–D) When subgrouped according to tumor size into p-T1a (CTNB group, n = 79; non-CTNB group, n = 78; p = 0.55, log-rank test), pT1b (CTNB group, n = 25; non-CTNB group, n = 51; p = 0.44, log-rank test), or p-T2a (CTNB group, n = 20; non-CTNB group, n = 68; p = 0.36, log-rank test), the differences in PFS became insignificant.

The incidence of adenocarcinoma was significantly higher in the CTNB group at 112 (90%), in comparison to the non-CTNB group at 161 (82%) (p = 0.04). Tumor size was significantly larger in the non-CTNB group (25±9 mm) in comparison to the CTNB group (19±9 mm) (p<0.001). The proportion of patients classified as pT1a was significantly higher in the CTNB group (64%) than in the non-CTNB group (40%) (p<0.001). In contrast, the proportion classified as p-T2a was significantly higher in the non-CTNB group (35%) than in the CTNB group (16%) (p<0.001). Regarding the surgical procedures, in the CTNB group, lobectomy was done in 111, and segmentectomy was done in 13 patients. In the non-CTNB group lobectomy was done in 183, bilobectomy was done in 3, and segmentectomy was done in 11 patients. Systematic lymph node sampling was done in all patients. The proportion of lobectomy or more was not significantly different between the two groups (p = 0.10). Percentages of the microscopic pleural, vascular, or lymphatic invasions were comparable between the two groups. The number of needle punctures in the CTNB group was 1 in 114 patients, 2 in 7, and 3 in 3 patients.

As for complications in the CTNB group, pneumothrax developed in 23 out of 124 patients (19%), and mild hemoptysis was seen in 12 out of 124 patients (10%), which all improved without any interventions. Needle-tract recurrences have not been detected so far in this group of patients.

### Recurrences

The follow-up periods in the two groups were comparable; median 45 months (range 11 to 93 months) in the CTNB group and median 42 months (range 3 to 100 months) in the non-CTNB group. Minimum follow-up was 6 months in patients without disease progression. The types of recurrence are summarized in Table 2. The overall proportion of recurrence was significantly higher in the non-CTNB group (35/197, 18%) than in the CTNB group (11/124, 9%) (p = 0.03). The rates of distant recurrences as well as local recurrences were both significantly higher in the non-CTNB group (28/197, 14% and 18/197, 9%, respectively) than in the CTNB group (8/124, 7% and 4/124, 3%, respectively) (p  = 0.03, 0.04, respectively). The pleural recurrences were ipsilateral in all patients. The rate of pleural recurrence tended to be higher in the non-CTNB group (7/197, 4%) than in the CTNB group (1/124, 1%), but this difference did not reach statistical significance. The rates of pleural recurrences alone were 1% in both groups (1/124 in the CTNB group, and 2/197 in the non-CTNB group, respectively).

### Pleural Recurrences

Kaplan-Meier analysis of pleural-recurrence free interval showed that there was no statistically significant difference between the two groups (Fig. 1). The difference in the proportions of pleural recurrence between the 2 groups was not significant (Table 2). The rates of pleural recurrences were also subgrouped according to tumor size, pT stage, microscopic pleural invasion, and histology (adenocarcinoma versus other histological types) (Table 3). The rates of pleural recurrences or pleural recurrences alone did not differ significantly between the CTNB and non-CTNB groups in any of the subgroups except in the microscopic pleural invasion (-) subgroup in which the recurrence rate was significantly higher in the non-CTNB group.

The details of 8 patients with pleural recurrences are individually summarized in Table 4. CTNB was performed only in patient no. 1; the other 7 patients were diagnosed by bronchoscopy. Pleural recurrence alone was seen in patient nos. 1–3. These 3 patients eventually died of local and distant disease progression. Other patients had concomitant distant recurrences. While the pleural recurrence may have been caused by CTNB in patient no.1, it could also have been due to the pleural invasion of the primary tumor.

### Progression-free Survival Rates

Overall PFS was significantly poorer in the non-CTNB group than in the CTNB group (Fig. 2A). the PFS in the CTNB group was not reduced in comparison to the non-CTNB group when patients were subgrouped according to pT stages (Fig. 2B–D).

## Discussion

In this particular study, the proportion of pleural recurrence was not significantly increased in the CTNB group in comparison to the non-CTNB groups. The overall proportion of pleural recurrence tended to be higher in the non-CTNB group than in the CTNB group, which was considered to be at least in part due to the larger tumor size in the former than in the latter. Three patients had pleural recurrences alone at first, 1 in the CTNB and 2 in the non-CTNB group. The proportions of pleural recurrences were not higher in the CTNB group in comparison to the non-CTNB group when subgrouped according to tumor size, or pT stage. The proportion of pleural recurrence was significantly higher in the non-CTNB group in the microscopic pleural invasion (-) subgroup. PFS was also not significantly different between the 2 groups when subgrouped by pT stages. These results are different from the previous reports showing increased incidences of pleural recurrences in early-stage lung cancer patients after transthoracic needle biopsies in comparison to other methods of diagnoses [10], [11]. The reason for this difference is not clear. Additionally, one of the previous reports usually conducted needle puncture twice per procedure [11], which may also have increased the rate of pleural recurrence. The incidence of complications was also within the previously reported ranges [13]–[15].

A limitation of the present study is that the median follow-up period of 45 months is relatively short in comparison to that of the previously reported studies, which were 60 months [11] and 80 months [10]. This may have resulted in the limited number of events, i.e., pleural recurrences. However, in the study by Matsuguma et al [10], the majority of pleural recurrences had appeared within 36 months of the procedure. We therefore consider that the median follow-up period of 45 months would be acceptable to evaluate the risk of pleural recurrence after CTNB. Currently, the minimum follow-up period in patients who are progression-free is 6 months. Therefore, it is possible that pleural recurrences will occur in this patient group with further follow-up, but in terms of the Kaplan-Meier estimate, the rate of pleural recurrence was not significantly different between the two groups at this point. Another major limitation of the present study is the sample size, and the difference in the potential selection bias between the CTNB and the non-CTNB groups. According to power analysis, assuming from the previous reports that pleural recurrence was approximately 6 times likely to occur in needle-biopsy patients [10], [11], the present study had enough sample size to evaluate this with a power of 80%. The actual difference was approximately 4 times in the present study (1% versus 4%), but still we consider that our study had moderate statistical power to detect this difference. To address the issue of selection bias, propensity score analysis based on a number of factors such as age, sex, tumor size, pathological stage, surgical procedure, observation period, and the presence of pleural invasion was attempted, but the number of events, in this case pleural recurrence, was considered to be too small in this particular study for the analysis to be valid. To this end, further accumulation of data is necessary. Nevertheless, the rate of pleural recurrence per se after CTNB was substantially lower in comparison to the previous reports, 1% in the present study versus 9% [10] and 6% [11], in the previous reports, respectively.

Collectively, at present, we do not consider that CTNB increases the risk of pleural recurrence in resectable lung cancer patients. However, it is also true that several case reports have noted cancer cell implantation along the biopsy route [8], [16]–[19]. Since prospective studies to address this issue would be difficult to design, accumulation of data from further retrospective studies will be necessary to clarify this point.
